# Atypical Features Resembling Poorly Differentiated Thyroid Carcinoma Presenting Entirely within a Follicular Adenoma

**DOI:** 10.1155/2018/7290343

**Published:** 2018-07-18

**Authors:** Daniel Ching, Connull Leslie

**Affiliations:** Department of Anatomical Pathology, PathWest, QEII Medical Centre, Western Australia, Australia

## Abstract

Poorly differentiated thyroid carcinoma (PDTC) is rare and is usually widely invasive at presentation. Here we present an unusual case with a component meeting diagnostic criteria for PDTC by Turin consensus proposal arising within a follicular adenoma. A 44-year-old female was found to have an incidental right thyroid nodule that was suggestive of follicular neoplasm on FNA. Histological examination of hemithyroidectomy revealed an 11 mm focus with insular growth pattern, alteration in cell morphology, and high mitotic count meeting criteria for PDTC. In addition there were several regions showing trabecular architecture with increased mitotic activity but not meeting criteria for PDTC. The literature for such cases is sparse but suggests much better prognosis than conventional invasive PDTC, although a biological potential for aggressive behaviour may be possible.

## 1. Introduction

Poorly differentiated thyroid carcinoma (PDTC) is rare and while some cases are recognised to arise from preexisting well differentiated follicular or papillary thyroid carcinomas, the tumours are usually widely invasive at presentation [1]. Here we present an unusual case in which a component meeting diagnostic criteria for PDTC by Turin consensus proposal [2] arises within a lesion otherwise appearing to represent a follicular adenoma.

## 2. Case Presentation

A 44-year-old female was found to have an incidental FDG-avid right thyroid lesion following staging PET for colorectal carcinoma. She was asymptomatic from the thyroid lesion and biochemically euthyroid. There was no personal or family history of thyroid disease and no prior history of radiation exposure to the head and neck region. CT scan of the neck confirmed a 40mm thyroid nodule, and ultrasound guided fine needle aspiration of this nodule was suggestive of a follicular neoplasm.

**Figure 1:**
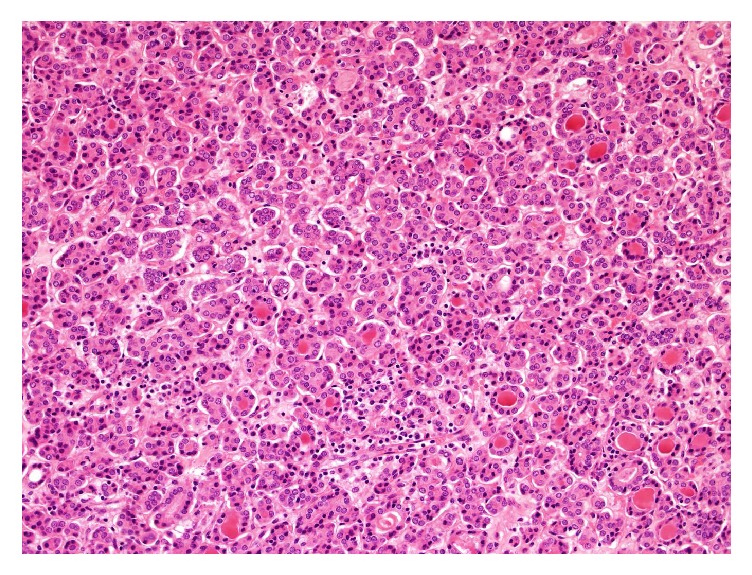
Background follicular adenoma: Microfollicles with scant colloid lined by cells with bland round to oval nuclei.

A right hemithyroidectomy was performed. The gross specimen weighed 67 grams and the cut surface revealed a round solid well-circumscribe tan nodule, with scant compressed residual thyroid parenchyma at the superior pole. The entire specimen was submitted for examination. Sections showed the nodule to be entirely encapsulated by a thick fibrous band without capsular or vascular invasion. The tumour showed predominantly areas in keeping with usual follicular adenoma formed by microfollicles with scant colloid and lined by cells with bland round to ovoid nuclei (Figure 1).

**Figure 2:**
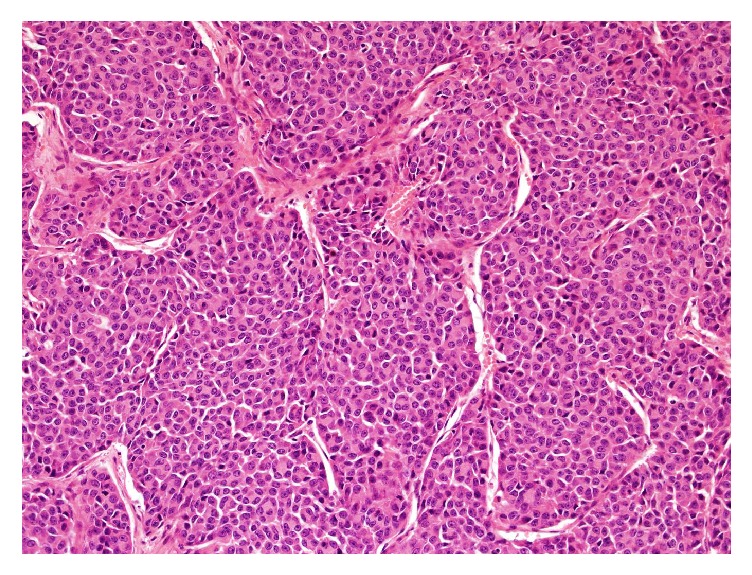
PDTC-like region with insular pattern and altered nuclear morphology.

**Figure 3:**
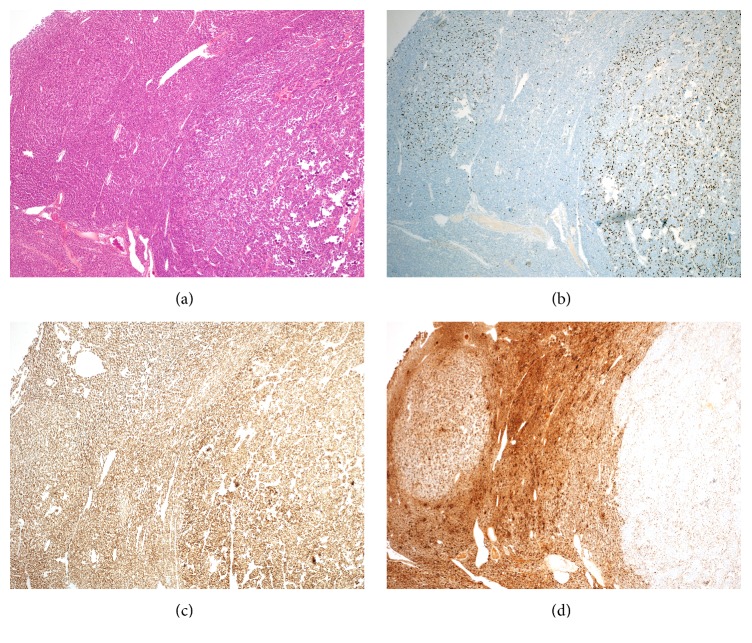
(A) Low power view of PDTC-like focus and adjacent trabecular region that does not fulfil PDTC criteria; (B) PDTC-like focus and adjacent trabecular region showing higher MIB-1 proliferation index; (C) all areas showing retained of TTF1; (D) PDTC-like focus showing loss of thyroglobulin expression and trabecular region with reduced expression.

Present centrally and entirely within the encapsulated and conventional adenoma was an 11 mm focus showing distinct insular growth pattern with atypical cell morphology. The cells contained round to ovoid nuclei with irregular nuclear membranes, small nucleoli, and high nuclear to cytoplasmic ratio. The mitotic count was very high (8/10 high power fields), although tumour necrosis was absent, meeting criteria for PDTC (Figure 2). In addition there were several regions, one of which was adjacent to the PDTC-like area, showing formation of trabeculae and high nuclear cytoplasmic ratio, without sufficient nuclear morphology or mitotic count for PDTC criteria (Figure 3(a)).

The PDTC-like focus and adjacent trabeculae region did show noticeably higher proliferation rate by MIB1 immunohistochemical (IHC) staining (Figure 3(b)). IHC staining showed retained expression of TTF1 (Figure 3(c)), with loss of thyroglobulin (which was retained in background follicular adenoma component and reduced expression in the adjacent trabeculae area) (Figure 3(d)). There was no expression of calcitonin, synaptophysin, chromogranin, or BRAFVE1 IHC staining. No nuclear features to suggest papillary thyroid carcinoma were present. Three lymph nodes excised with the thyroidectomy were negative for malignancy.

The background thyroid parenchyma showed patchy lymphocytic aggregates suggestive of lymphocytic thyroiditis. After multidisciplinary discussion, the patient proceeded to have a left completion thyroidectomy which was negative for adenoma or malignancy and also showed features of lymphocytic thyroiditis. Follow-up 12 months after resection shows patient is alive and well.

## 3. Discussion

Follicular adenoma is a benign lesion [1], defined by complete encapsulation and absence of vascular invasion, which usually has a microfollicular, solid, or trabecular architecture, although insular patterns may be present. Nuclear features should be bland with smooth nuclear contours and mitotic figures being rare.

The WHO definition of PDTC refers to the Turin proposal [2] which requires the tumour to be of follicular cell derivation by conventional criteria and have a solid/trabecula/insular growth pattern, with absence of papillary thyroid carcinoma features and having features of either convoluted nuclei, 3 or more mitoses per 10 high power fields, or tumour necrosis [1, 2]. Usually, and noted in this system, PDTC will have “conventional diagnostic features for malignancy”, that is, either capsular or vascular invasion.

The significantly altered architectural and cytological features, in addition to significant mitotic activity and localised loss of expression of thyroglobulin, suggest that a component of this lesion has acquired potentially aggressive biological potential although under strict application of Turin consensus lack of capsular or venous invasion may preclude this diagnosis.

Features of PDTC without capsular or vascular invasion are rare although described. Some studies have suggested that the presence of focal areas resembling PDTC in an otherwise well differentiated tumour may be associated with aggressive features and poor prognosis. Bongiovanni et al. reported an encapsulated, nonangioinvasive follicular thyroid neoplasm with high grade features (insular pattern in 10% of lesion, up to 14 mitoses/10 HPF and necrosis) which subsequently developed multiple metastases [3]. Other studies have demonstrated no recurrence or metastasis after extensive follow-up. Rivera et al. followed 6 examples of encapsulated, nonangioinvasive follicular thyroid neoplasms with high grade features without recurrence or metastasis with a median follow-up of 11.9 years [4]. Hiltzik et al. described 4 examples without recurrence or metastasis with a follow-up that reached up to 11 years [5]. In a review of 111 cases Decaussin et al. noted 6 cases with distant metastases but without vascular and/or capsular invasion, although in all these cases sampling was not complete [6].

The proportion of PDTC present in encapsulated lesions without capsular and/or vascular invasion reported in the literature is unclear. Dettmer et al. reported that focal presence of PDTC features, as little as 10%, in an otherwise well differentiated tumour may be associated with aggressive features and/or unfavourable prognosis [7]. In our case, PDTC-like areas accounted for an estimated 10% of the nodule volume.

PDTC, earlier known as insular carcinoma, is considered to have intermediate prognostic implication (between well differentiated and anaplastic carcinoma) [8, 9]. Literature discussing the entity is often problematic as histologic definition varies between studies.

When well defined as an invasive malignancy PDTC has a 5-year survival rate ranging from 60 to 70%, with recurrences usually presenting within 3 years [8, 10]. The reported rate of metastases from well differentiated thyroid cancers is estimated to range from 9% to 15%. Insular component was found in >50% of cases with metastases. The estimated risk of distant metastases was 17x higher in the presence of an insular component [6]. Whether these data could be extrapolated to cases with PDTC features in a noninvasive lesion is unclear.

Given that PDTC can arise from well differentiated carcinomas, during early stages the dedifferentiated component could be assumed to initially appear encapsulated and noninvasive. Dedifferentiation confers a worse prognosis, in part due to less responsiveness to radioiodine treatment [11]. Interestingly there were also separate trabecular foci with increased mitotic activity and proliferation rate by MIB1 staining, although without sufficient cytological features to be considered PDTC-like.

The literature would suggest much better prognosis than conventional invasive PDTC, although a biological potential for aggressive behaviour may be possible.
